# The effect of ultralow-dose antibiotics exposure on soil nitrate and N_2_O flux

**DOI:** 10.1038/srep16818

**Published:** 2015-11-26

**Authors:** Stephanie L. DeVries, Madeline Loving, Xiqing Li, Pengfei Zhang

**Affiliations:** 1 Department of Earth and Atmospheric Sciences, City College of New York, 160 Convent Avenue, New York, NY, 10031, USA; 2 Department of Earth and Environmental Sciences, Graduate School and University Center, City University of New York, 365 5^th^ Avenue, New York, NY, 10016, USA; 3 Laboratory of Earth Surface Processes, College of Urban and Environmental Sciences, Peking University, Beijing 100871, China

## Abstract

Exposure to sub-inhibitory concentrations of antibiotics has been shown to alter the metabolic activity of micro-organisms, but the impact on soil denitrification and N_2_O production has rarely been reported. In this study, incubation and column transport experiments were conducted on soils exposed to as many as four antibiotics in the ng·kg^−1^ range (several orders of magnitude below typical exposure rates) to evaluate the impact of ultralow dose exposure on net nitrate losses and soil N_2_O flux over time. Under anaerobic incubation conditions, three antibiotics produced statistically significant dose response curves in which denitrification was stimulated at some doses and inhibited at others. Sulfamethoxazole in particular had a stimulatory effect at ultralow doses, an effect also evidenced by a near 17% increase in nitrate removal during column transport. Narasin also showed evidence of stimulating denitrification in anaerobic soils within 3 days of exposure, which is concurrent to a statistically significant increase in N_2_O flux measured over moist soils exposed to similar doses. The observation that even ultralow levels of residual antibiotics may significantly alter the biogeochemical cycle of nitrogen in soil raises a number of concerns pertaining to agriculture, management of nitrogen pollution, and climate change, and warrants additional investigations.

A significant portion of antibiotics administered to humans and livestock are excreted as active, non-metabolized compounds^1^. When manure, sewage sludge, wastewater, or contaminated surface waters are applied to soils, these are conveyed to the soils where they often persist and remain bioavailable. The maximum concentration of antibiotics transferred to soil is often within the μg·kg^−1^ to mg·kg^−1^ range where a number of studies have shown that delayed or reduced rates of denitrification may result and thus have direct consequences for non-point source N_2_O or NO_3_^−^ pollution^2^,^3^,^4^,^5^. Far less is known about the effects of antibiotics at lower exposure levels. How and to what magnitude minimum exposure levels, including those that may fall below analytical detection limits, impact the structure and function of soil microbial communities has rarely been considered. The primary objective of this research was to evaluate whether ultra-low (ng·kg^−1^) exposure to environmentally relevant antibiotics affects total nitrate losses and/or N_2_O flux over time. The antibiotics selected for study include narasin (NAR), an ionophore active against many gram-positive bacteria^6^, gentamicin (GTC), an aminoglycoside that targets gram-negative bacteria and some facultative anaerobes^7^, and two broad-spectrum^8^ sulfonamides, sulfamethoxazole (SMX) and sulfadiazine (SDZ). NAR and GTC are both approved in the United States for use in poultry production and the residual antibiotic concentration prior to field application may range from 10–10,000 μg kg^−1^ litter^9^. Based on a 9200 kg·acre^−1^ litter application rate and a 15 cm plow depth^10^, the quantity of antibiotic transferred to soil may be as low as 100 ng·kg or as high as 100 μg·kg^−1^. Considered medically important, both SMX and SDZ have restricted application in animal husbandry in the United States^11^ but are still in use elsewhere and are often detected in sewage sludge and wastewater. SMX and SDZ are also among the most frequently detected antibiotics in groundwater with reported concentrations ranging from 0.08 ng·L^−1^ 12 to 1.11 μg·L^−1^ 13. Assuming a partition coefficient (Kd) of 2.0 L•kg-1^14^, the concentration in saturated soils can be estimated between 0.16 ng·kg^−1^ and 2.22 μg·kg^−1^ though this may vary depending upon the antibiotic source (e.g., sewage sludge vs. groundwater) and is subject to rapid dissipative losses^15^. The effect of all four selected antibiotics on gross denitrification was measured in terms of nitrate losses from anaerobic pot incubations in which soils were exposed to ng·kg^−1^ doses. NAR and SMX generated the strongest responses and were selected for additional study. SMX is among the few veterinary antibiotics shown to leach into the saturated zone^16^ and was therefore chosen for saturated column experiments. N_2_O flux experiments, conducted over moist soils, were performed using NAR, which is less mobile^17^ and tends to sorb in the upper, temporally moist soil horizons where N_2_O is easily lost to the atmosphere.

## Results and Discussion

### Anaerobic nitrate reduction

**Figure 1:**
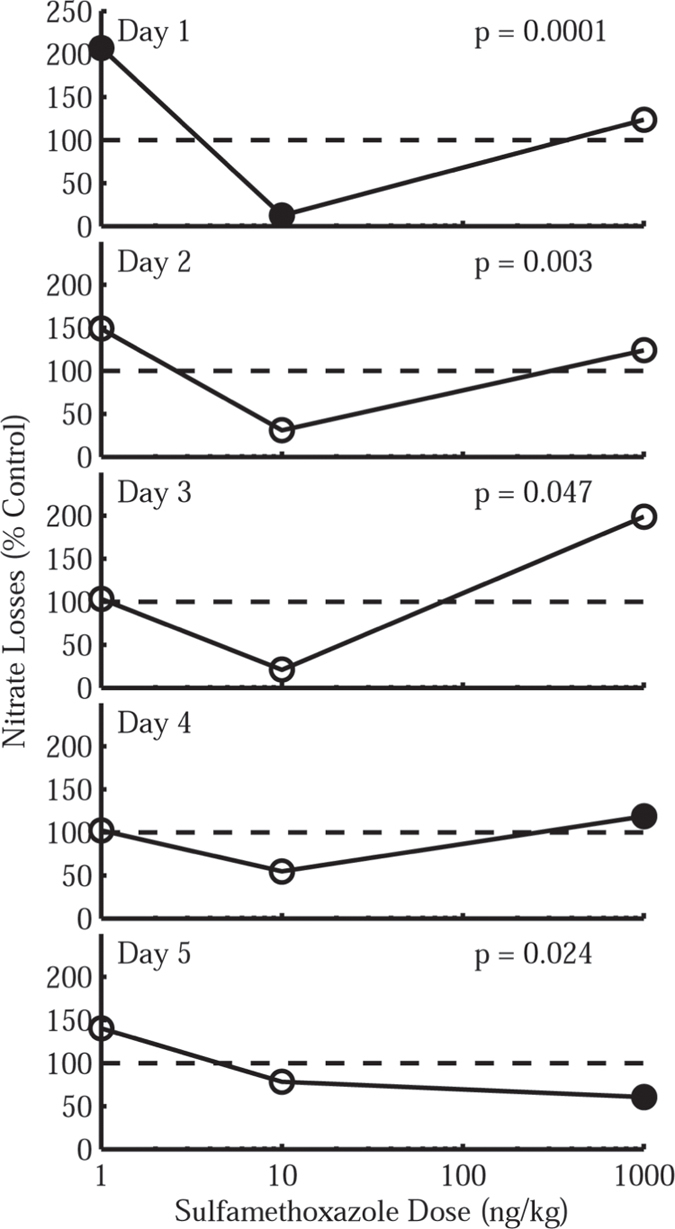
Time-Dose Response curves illustrating the percentage of extractable nitrate lost relative to the control in soils treated with sulfamethoxazole. Results shown are the average of three replicates collected at each sampling period. Values above 100% (dashed line) indicate that nitrate losses are stimulated relative to the control whereas values less than 100% point to nitrate losses inhibited relative to the control.

KNO_3_ solutions with various low doses of selected antibiotics (SMX, SDZ, NAR, and GTC) were added to pre-incubated soils, incubated, and extractable nitrate was determined (see Materials and Methods for details). All four antibiotic treatments yielded some combination of stimulated (% Control > 100%) and inhibited nitrate losses (% Control < 100%) and exhibited a temporal trend toward inversion, e.g., early stimulation followed by inhibition after longer incubation periods (see Table 1). Analysis of variance (ANOVA) identified statistically significant dose-responses in for 3 of the 4 antibiotics tested (see Table 2); the majority of these were observed in soils treated with SMX. Figure 1 illustrates the time-dose response (in terms of percentage of extractable nitrate lost relative to the control) in soils treated with SMX. Four statistically significant, U-shaped dose response curves (p < 0.05) in which nitrate losses initially exceed that of the control at the lowest (1 ng·kg^−1^, 207%) and highest (1000 ng·kg^−1^, 123%) doses but are inhibited relative to the control at 10 ng·kg^−1^ (12%) are observed. This overall pattern is maintained for a total of 4 days, after which the magnitude of both stimulation and inhibition decline. On Day 5, only the 1 ng·kg^−1^ dose corresponds to stimulated nitrate losses. Treatment with SDZ, NAR, and GTC resulted in far less distinct time-dose-response patterns, but showed an overall tendency for the rate of nitrate removal to increase as a result of exposure (Table 1). Where SDZ was applied, no individual dose-response was determined to be statistically significant (Table 2), but a general pattern of accelerated nitrate losses were observed at one or more sampling points for all four doses (Table 1). These were most commonly observed on Days 1 and 2 and the lowest dose (1 ng·kg^−1^) yielded a stimulatory effect for 4 of the 5 days tested. In soils treated with NAR, all four doses stimulated nitrate losses on Day 1 and Day 3 and all resulted in a diminished removal rate on Day 5 (Table 1). Three of these doses (1, 10, and 1000 ng·kg^−1^) were observed to correspond with increased nitrate removal rate on all but the 5^th^ day of sampling. Both the maximum stimulation (1000 ng·kg^−1^, 199%) and a significant dose-response occurred on Day 3 (p = 0.02, Table 2). Higher doses of GTC (100 ng·kg^−1^ and 1000 ng·kg^−1^) also stimulated nitrate removal for four of the five days tested (Table 1). Though stimulation of the greatest magnitude occurs on Day 2 (144%, 100 ng·kg^−1^), a statistically significant dose response does not emerge until Day 4 (Table 2), where inhibition observed at 1 and 1000 ng·kg^−1^ contrasts with stimulation the two middle doses (10 and 100 ng·kg^−1^).

The results of these anaerobic denitrification experiments provide evidence that ecologically significant microbial communities in soil and sediment may have a statistically significant dose-response when exposed to antibiotics at ultra-low concentrations (ng·kg^−1^). The most frequently observed effect was an accelerated loss of soil nitrate, which stands in contrast to expectation because antibiotics are generally employed to inhibit microbial activity. Based upon broad temporal trends exhibited by these results (stimulation observed in 63% of samples on Days 1–4 and 75% inhibited on Day 5) and the distinctive U-shaped dose-response curve corresponding to SMX treatment, it is tempting to draw some comparison between these outcomes and direct stimulation hormesis (Figure S1) in which sub-inhibitory exposure to a toxin can produce a stimulatory effect in the target organism^18^. Though it is possible that hormetic responses can and do occur in soils exposed to these antibiotics, any apparent hormetic effect is likely the result of population-level consequences resulting from individual hormesis and not the hormetic response in and of itself. An alternate and perhaps simpler hypothesis is that accelerated nitrate reduction is the functional outcome of selective antibiotic pressure within the more complex soil microbial community. For example, NAR is active against gram-positive bacteria and since most denitrifying organisms are gram-negative^19^, NAR is unlikely to inhibit or stimulate growth or enzymatic activity within this functional group. On the other hand, inhibition of one or more gram-positive organisms in the soil microbial community is expected and may increase the availability of resources to competing organisms, including the gram-negative denitrifiers, allowing them to grow at the expense of inhibited species.

Evidence that both broad-spectrum and gram-positive/gram-negative antibiotics can and do affect the structure and function of soil microbial communities at higher doses (mg·kg^−1^) is abundant^20^. Of the antibiotics tested in the present study, for example, SDZ has been reported to decrease microbial diversity^21^ and to increase the ratio of ammonia oxidizing archaea to ammonia oxidizing bacteria^22^. At comparable doses, SDZ and SMX^23^,^24^ have both been observed to increase the ratio of fungi to bacteria in soils. Differences in antibiotic agency, i.e., broad-spectrum vs. gram negative/positive, between different antibiotics can be expected to impact the microbial population differently and may account for variations in the overall dose-time-response curves reported here but does not explain why maximum stimulation in the sulfonamides corresponds to the lowest doses (1 ng·kg^−1^) but occurs in NAR and GTC-treated soils at higher doses (1000 ng·kg^−1^ and 100 ng·kg^−1^, respectively).

### Denitrification in saturated sediment columns

**Figure 2:**
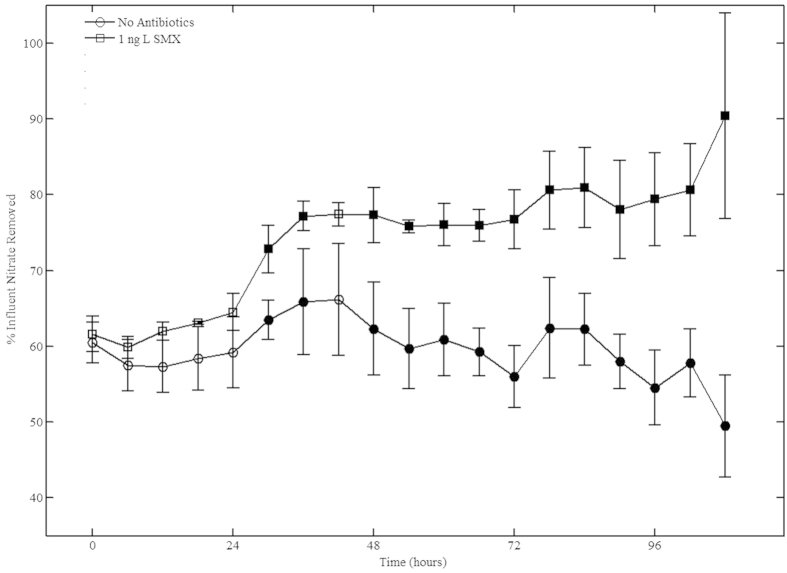
Percent influent nitrate removed from control (o) and experimental (☐) columns during transport through saturated soil columns receiving a continuous flow of nitrate nitrogen and glucose. Experimental columns were spiked with 1 ng·L^−1^ SMX from t = 24 to t = 108. Triplicate columns were run for the spiked as well as the control tests. Statistically different nitrate reduction (p < 0.05) was observed from t = 30 to t = 108 and is indicated with solid markers.

Where the effects of antibiotics on soil function have been evaluated, denitrification has consistently been shown to be inhibited where higher doses of antibiotics (>500 μg·kg^−1^) were administered to soil^2^, sediment^5^,^25^,^26^ and groundwater^3^,^27^. The consistency of these results contrast greatly to the combined stimulation and inhibition reported here for ng·kg^−1^ doses in anaerobic soils and further to the results of anaerobic column experiments. Figure 2 illustrates effluent nitrate concentration (as a % of influent concentration) for a set of six columns receiving a 1 mM nitrate influent solution. Starting from t = 24 hours, 1 ng·L^−1^ SMX was continuously added to the influent of three of these columns. Prior to the addition of SMX, approximately 60% of influent nitrate was reduced during transit through each of the six columns. As the experiment continued, nitrate reduction in the three control columns showed slight diurnal variations, possibly resulting from temperature changes in the laboratory, but the overall average remained relatively constant at ~60%. In contrast, the columns receiving influent spiked with 1 ng·L^−1^ SMX showed an increase in overall nitrate reduction, with total nitrate losses increasing from an initial 60% to nearly 90% at the end of the experiment. According to student t-tests, this increase is statistically significant at or above the 95% confidence level from t = 30 through the end of the experiment (see Supplementary Information). Unlike the anaerobic incubation experiment where the maximum stimulatory effect of SMX was observed on Day 1, stimulation in the column experiments appears to steadily increase over time. The discrepancy between these results may indicate that the stimulatory effect of SMX at the 1 ng·kg^−1^ or 1 ng·L^−1^ level is reduced over time by biodegradation. The soil used for the anaerobic incubation experiment received only a single dose of SMX at the beginning of the experiment whereas the columns received a steady supply of SMX-spiked influent that was prepared daily. The gradual increase in denitrification rate relative to the control might indicate that any microbial shift resulting from 1 ng·L^−1^ SMX exposure is both maintained and enhanced by continued antibiotic pressure at this dose.

### N_2_O Flux

**Figure 3:**
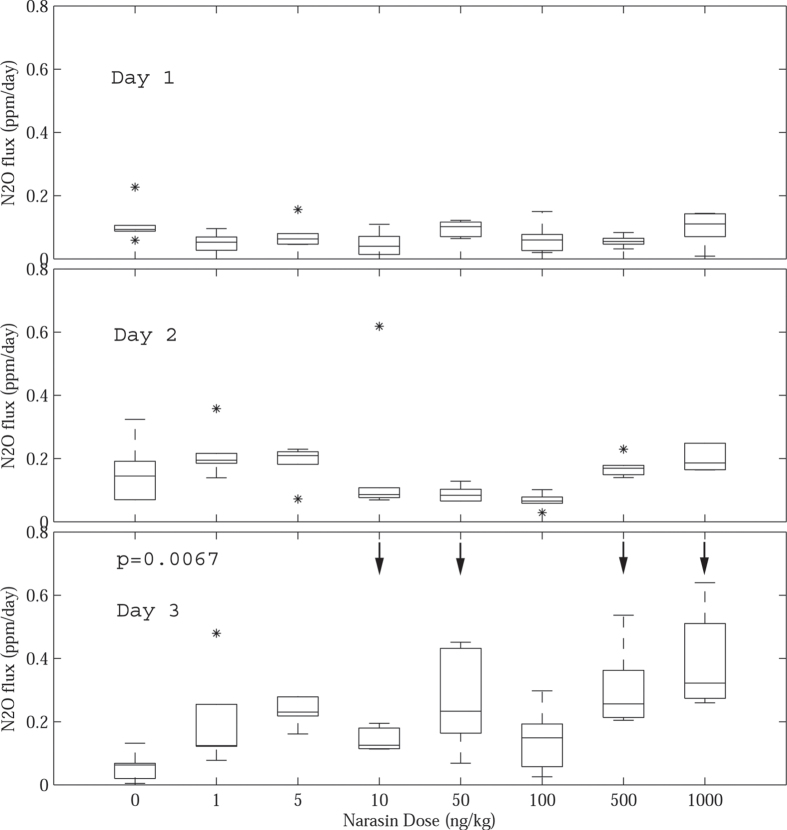
Box-whisker plot of daily N_2_O flux (ppm·day^−1^) in moist soil (40% water filled pore space) treated with 0–1000 ng·kg^−1^ Narasin. For each dose, 6 replicate samples were analyzed; statistical outliers are shown as asterisks and data that differ significantly from the control are indicated with arrows.

Where any changes in denitrification rate or potential in soil and sediment are observed, changes in the flux rate of N_2_O, a powerful greenhouse gas are also likely. Though at least one previous study has reported a decrease in N_2_O from mineral soils treated with 1-1000 μg·L^−1^ SMX^5^, the opposite effect was observed in moist soils treated with 1-1000 ng·kg^−1^ NAR. As seen in Fig. 3, the average N_2_O flux is around 0.1 ppm·day^−1^ for all antibiotic treatments and the control after only one day of incubation, but on Day 3 a statistically significant dose-response emerged (see Table 3, p = 0.0067). The dose-response observed is nearly linear with N_2_O flux ranging from 0.1 ppm·day^−1^ (Control) to approximately 0.4 ppm·day^−1^ (1000 ng·kg^−1^). Although NAR was also shown to stimulate nitrate reduction at each of these doses on Day 3 (Table 1), it is unlikely that accelerated denitrification alone accounts for the increase in N_2_O flux, especially at the highest dose where nitrate losses are 200% of the control but net N_2_O flux are 300%. Surplus N_2_O flux may result from either a shift in the N_2_O:N_2_ ratio, a mechanism suggested by Hou *et al.* (2015) whose experiments with 0.05–100 μg·L^−1^ sulfamethazine in sediment showed an increase in N_2_O despite inhibited denitrification^26^, or it may indicate that antibiotics also affect nitrifier-denitrification rates (NH_2_OH → N_2_O or NO_2_^−^ → NO → N_2_O) in aerobic soils^28^. To better constrain source of increased N_2_O flux, future studies would benefit from the use of isotopic tracers that can be used to distinguish between N_2_O sources^28^.

## Conclusions

Disturbances to the biogeochemical nitrogen cycle have been reported in soils and sediment exposed to a wide-range of antibiotic compounds. The effects observed at both ultralow (ng·kg^−1^) and moderate (μg·kg^−1^) antibiotic concentrations include shifts in microbial diversity and community structure as well as overall function, which raises a number of concerns pertaining to agriculture, nitrogen management, and climate change. In agriculture, factors controlling microbial N-cycling are well-characterized and the resulting relationships have been used to develop a number of different modeling tools to improve nitrogen use efficiency and reduce nitrogen loading rates to sensitive ecosystems^29^,^30^. At present, these models do not take into account potential temporal and functional shifts in the biogeochemical nitrogen cycle that may arise when soil microorganisms are exposed to antibiotics.

Natural mitigation of aquatic nitrate pollution, which is tied to a number of human health risks^31^ and to the degradation of aquatic ecosystems^32^,^33^ may also be affected. Excess nitrate leached from soil is significantly reduced during transport through soil and sediment with denitrification (NO_3_^−^ → N_2_O → N_2_) estimated to reduce groundwater NO_3_^−^ by as much as 50% on a watershed scale^34^. Denitrification is inhibited by a number of antibiotics when the dose exceeds 500 μg·kg^−1^, which is distinctly negative outcome in terms of water quality and the health of aquatic ecosystems, but may be stimulated for up to 3 or 4 days when soils are exposed to <1 μg·kg^−1^ SMX, SDZ, NAR, or GTC. A stimulated response at physically and biologically reduced concentrations might partially counter high-dose inhibition by enhancing denitrification over longer, low-dose exposures, but appears to have the potential to increase microbial production of nitrous oxide (N_2_O), a powerful greenhouse gas and the leading modern contributor to stratospheric ozone depletion^35^. Whether these pathways or anaerobic methane (CH_4_) production may also be stimulated by exposure to ultralow doses of antibiotics is presently unknown, but is very relevant to climate research. Based upon the growing body of evidence suggesting that both low and high dose antibiotics in the terrestrial environment can and do affect ecologically important aspects of the biogeochemical nitrogen cycle, additional research is strongly encouraged to include: (1) a larger number of antibiotics tested at both low (ng·kg^−1^) and high (μg·kg^−1^) exposure levels, (2) a wide variety of different soils and sediments (3) use of isotopic tracers to better constrain N_2_O source where denitrification and nitrification are affected, and (4) chronic and/or repeat exposure tests to determine whether single-dose effects are persistent and/or cumulative and the role of antibiotic resistance in those changes.

## Materials and Methods

### Statistical Analysis

Student t-tests were used to evaluate the statistical significance of individual treatments relative to the control at each sampling point (95% confidence interval) and an analysis of variance (ANOVA) was used to determine whether dose-responses (C/C_0_) were statistically significant at the 95% confidence level. Comparison of group means with multiple t-tests would lead to significant Type-1 errors (e.g., 14.3% for 3 t-tests) whereas the Type-1 errors remain at 5% in one way ANOVA analysis of multiple group means^36^.

### Soil Sampling

The soil used in this study was sampled from a coastal farm in (Bull’s Eye Farm) along the Upper Indian River Bay, near Milford, Delaware. The history of the site is known beyond 20 years by personal communication with the farmer who leases the land and the authors are assured that the soils have not previously been exposed to antibiotics. Groundwater sampling conducted at this site in 2012 corroborates this conclusion (unpublished data). Sandy loam topsoil and a sandy subsoil Topsoil samples (sandy loam) were composited from 10 cm cores, air-dried, sieved to 2 mm, and stored at 4 °C. The subsoil (sandy) was collected from the saturated zone at 2 meters depth using an auger. Following collection, the samples were air-dried and stored at 4 °C.

### Anaerobic Incubation Experiment

A set of 48 soil samples (10 grams each, air-dry basis) were treated with 10 mL of 12.5 mg/mL glucose solution and then pre-incubated at 25 °C in 50 mL centrifuge tubes in order to establish anaerobic conditions and deplete residual nitrate from the soil. Extractable nitrate was confirmed to be zero after 9 days. The pre-incubated samples were then dosed with 125 mg glucose, 100 mg KNO_3_ and 1, 10, 100, or 1000 ng·kg^−1^ narasin, gentamicin, sulfadiazine, or sulfamethoxazole under N_2_ gas as a 1 mL solution. Each treatment was performed in triplicate, with control samples receiving no antibiotic. Following amendment, the topsoil samples were incubated in the dark at 25 °C for an additional 1–5 days and then extracted with 10 mL of 1 M KCl. The extractable nitrate was quantified using a SEAL AQ2 Discrete Nutrient Analyzer (Seal Analytical, Mequon, Wisconsin, USA).

### N_2_O Flux Experiment

75 g air-dried soil was measured into 144 polypropylene containers (4 cm × 4 cm × 6 cm) and moistened with 10 mL Milli-Q water. The containers were capped and pre-incubated at room temperature for 4 days. Following the pre-incubation period, the soils were treated with an antibiotic solution (0, 1, 5, 10, 50, 100, 500, or 1000 ng·kg^−1^ Narasin final concentration) and a nutrient solution (34 mg·mL^−1^ (NH_4_)_2_SO_4_ and 21 mg/mL KNO_3_). Additional Milli-Q was added to raise the total moisture content to 40% Water-Filled Pore Space (WFPS) and the containers were placed inside 500 mL Kilner Jars outfitted with two gas-tight sampling ports. Headspace samples were collected from 6 replicates for each treatment at 24, 48, and 72 hours after the addition of antibiotic and nutrient solutions. Samples were transferred to evacuated Exetainer vials and analyzed by Isotope Ratio Mass Spectrometry at the University of California Davis.

### Column Experiment

A set of six 15 × 2.5 cm (length × diameter) glass columns were packed with air-dried sandy subsoil. The columns were purged with CO_2_ for 20 minutes and then saturated bottom to top with degassed Milli-Q water. All six columns underwent a two week pre-treatment during which a nutrient solution containing 0.5 mM NO_3_^−^ and 0.4 mM glucose (Control) was continually passed through the columns at an average linear velocity of 1 m/day. After 2 weeks, effluent samples were collected in 6 hour increments. Twenty-four hours after the first fractions were collected the influent to three columns (Experimental) was modified by the continuous addition of 1 ng∙L^−1^ sulfamethoxazole. The influent vessel, all tubing, and the columns were wrapped in aluminum foil to prevent photodegradation of the antibiotic during transit and additional fractions were collected for 3.5 days following the initial addition of antibiotic to the experimental columns. The nitrate concentration of effluent samples was determined using ion chromatography with an AS14A 5-μm column (Dionex, Waltham, Massachusetts, USA).

## Additional Information

**How to cite this article**: DeVries, S. L. *et al.* The effect of ultralow-dose antibiotics exposure on soil nitrate and N_2_O flux. *Sci. Rep.* **5**, 16818; doi: 10.1038/srep16818 (2015).

## Supplementary Material

Supplementary Information

## Figures and Tables

**Table 1:** Percentage of extractable nitrate lost relative to the control in soils treated with sulfamethoxazole, sulfadiazine, narasin, and gentamicin.

	Dose (ng·kg^−1^)	Day 1	Day 2	Day 3	Day 4	Day 5
SMX	1	207* (58)	**149** (35)	13 (50)	**102** (24)	**140** (70)
	10	12* (26)	31 (39)	21 (42)	55 (32)	78 (29)
	1000	**124** (28)	**124** (30)	**199** (81)	119* (32)	60* (31)
SDZ	1	**125** (31)	**128** (20)	**124** (43)	**106** (28)	37* (60)
	10	**118** (43)	**105** (23)	86 (37)	77 (18)	42 (39)
	100	**109** (27)	98 (19)	97 (40)	76 (38)	**104** (44)
	1000	57 (64)	86 (48)	**109** (35)	83 (21)	74 (37)
NAR	1	**106** (39)	**113** (22)	**117** (46)	**105** (25)	71 (29)
	10	**127** (61)	**112** (27)	**126** (45)	**104** (29)	77 (31)
	100	**120** (41)	96 (25)	**117** (38)	68 (33)	82 (30)
	1000	**124** (28)	**124** (30)	199* (81)	**119** (32)	60* (31)
GTC	1	75 (24)	**111** (42)	78 (39)	90 (21)	62 (52)
	10	65 (67)	90 (21)	59 (96)	**107** (25)	**100** (36)
	100	134* (32)	**144** (22)	**115** (38)	**122** (29)	78 (40)
	1000	**113** (41)	**107** (15)	**118** (38)	72 (17)	**107** (37)

Results shown are the average of three replicates collected at each sampling period with standard error shown in parentheses. Values above 100% (shown in bold) indicate that nitrate losses are stimulated relative to the control whereas values less than 100% point to nitrate losses inhibited relative to the control. Individual treatments deemed by a student t-test to be statistically different (p < 0.05) from the control are denoted with an asterisk.

**Table 2:** Results of One-Way ANOVA for soils treated with 1, 10, 100, or 1000 ng·kg^−1^ sulfamethoxazole, sulfadiazine, narasin, and gentamicin over a five-day sampling period.

		Day 1	Day 2	Day 3	Day 4	Day 5
SMX	F(3,8)	29.82	11.05	4.15	3.11	5.43
	P value	**0.0001**	**0.003**	**0.047**	0.087	**0.024**
SDZ	F(4,10)	1.75	1.16	1.21	1.47	1.99
	P value	0.21	0.39	0.367	0.28	0.17
NAR	F(4,10)	0.400	0.83	4.81	2.72	1.35
	P value	0.80	0.53	**0.02**	0.09	0.31
GTC	F(4,10)	1.88	2.66	0.88	8.68	1.13
	P value	0.19	0.09	0.51	**0.002**	0.39

The F-statistic was calculated for concentration of nitrate measured in triplicate samples grouped by dose. Dose-response relationships are deemed statistically significant where F_stat_ > F_crit_. P-values less than 0.05 are shown in bold.

**Table 3:** Results of One-Way ANOVA for N_2_O flux from Narsin-treated soils.

		Day 1	Day 2	Day 3
Narasin	F(7,40)	2.11	1.73	3.34
	P value	0.06	0.12	**0.0067**

The F-statistic was calculated for the N_2_O flux measured in six replicate samples grouped by dose. Dose-response relationships are deemed statistically significant where F_stat_ > F_crit_. P-values less than 0.05 are shown in bold.
