# Supplementary material for: The effect of ultralow-dose antibiotics exposure on soil nitrate and N2O flux
Source: Sci Rep. 2015 Nov 26;5:16818. doi: 10.1038/srep16818 (PMC4660347; doi:10.1038/srep16818)
Supplement: Supplementary Information [file srep16818-s1.doc]

**Supplemental Information**

**The effect of ultralow-dose antibiotics exposure on soil nitrate and N_2_O flux**

Stephanie L. DeVries^a,b^, Madeline Loving^a^, Xiqing Li^c^, and Pengfei Zhang^a,b*^

^a^Department of Earth and Atmospheric Sciences, City College of New York,

160 Convent Avenue, New York, NY, 10031, USA

^b^Department of Earth and Environmental Sciences, Graduate School and University Center,

City University of New York, 365 5^th^ Avenue, New York, NY, 10016, USA

^c^Laboratory of Earth Surface Processes, College of Urban and Environmental Sciences,
Peking University, Beijing 100871, China

______________________________________________________

^*^Corresponding author:

Tel: +1 212 650 5609; fax: +1 212 650 6482; E-mail address: pzhang@ccny.cuny.edu.

Figure S1. Dose-Time-Response characteristics of direct stimulation hormesis. Time 1, Time 2, and Time 3 do not reference a specific unit of time but indicate a time-based progression during which low doses of a toxin or inhibitor may initially lead to stimulated activity, followed by a gradual, time-dependent shift toward inhibited activity at the same dosages. Adapted from Calabrese and Baldwin.^1^


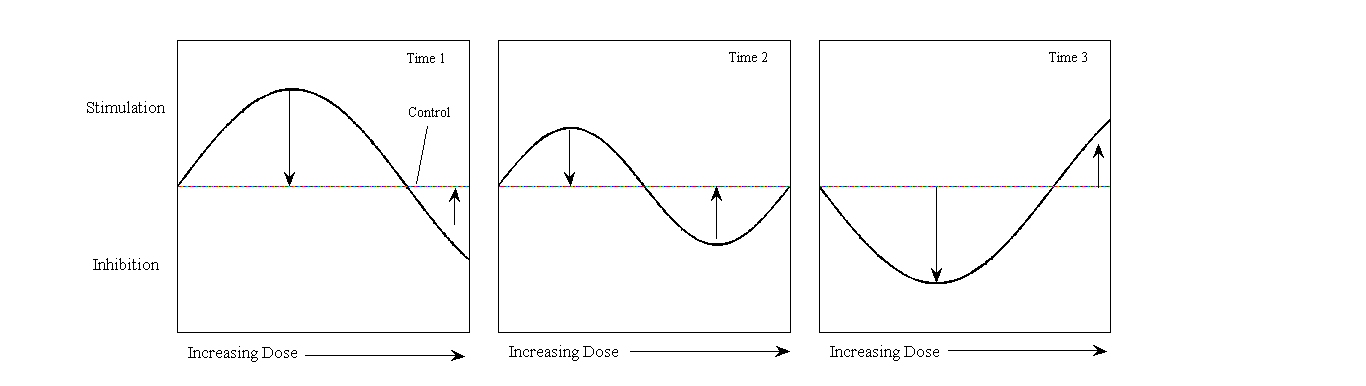


Table S1. Results of Student t-test for effluent nitrate in SMX-treated soils vs. untreated soils. P-values less than 0.05 for paired data are shown in bold.

| **Time (hours)** | **P-value** |
| --- | --- |
| 0 | 0.345 |
| 6 | 0.182 |
| 12 | 0.054 |
| 18 | 0.097 |
| 24 | 0.162 |
| **30** | **0.037** |
| **36** | **0.047** |
| 42 | 0.064 |
| **48** | **0.049** |
| **54** | **0.016** |
| **60** | **0.003** |
| **66** | **0.002** |
| **72** | **0.001** |
| **78** | **0.015** |
| **84** | **0.001** |
| **90** | **0.016** |
| **96** | **0.015** |
| **102** | **0.009** |
| **108** | **0.005** |

Table S2. Mean, Standard Deviation (parentheses), and significance levels (italics) determined by student’s t-test for paired means (relative to control) for N_2_O flux from in aerobic soils treated with 1-1000 ng·kg^-1^ Narasin.

| **Dose (ng/kg)** | **Day 1** | **Day 2** | **Day 3** |
| --- | --- | --- | --- |
| **1** | 0.048 | 0.198 | 0.141 |
|  | **(0.007)** | (0.082) | (0.075) |
| **5** | 0.060 | 0.188 | 0.224 |
|  | **(0.008)** | (0.198) | (0.085) |
| **10** | 0.044 | 0.193 | 0.142 |
|  | **(0.002)** | (0.453) | **(0.002)** |
| **50** | 0.096 | 0.043 | 0.263 |
|  | (0.629) | (0.070) | **(0.032)** |
| **100** | 0.070 | 0.066 | 0.145 |
|  | **(0.003)** | (0.432) | (0.061) |
| **500** | 0.056 | 0.173 | 0.305 |
|  | (0.067) | (0.413) | **(0.007)** |
| **1000** | 0.098 | 0.368 | 0.388 |
|  | (0.548) | (0.120) | **(0.006)** |

**References:**

1 Calabrese, E. J. & Baldwin, L. A. Defining hormesis. *Human and Experimental Toxicology* **21**, 91-97 (2002).
